# BRCA-DIRECT digital pathway for diagnostic germline genetic testing within a UK breast oncology setting: a randomised, non-inferiority trial

**DOI:** 10.1038/s41416-024-02832-2

**Published:** 2024-10-01

**Authors:** Bethany Torr, Christopher Jones, Grace Kavanaugh, Monica Hamill, Sophie Allen, Subin Choi, Alice Garrett, Mikel Valganon-Petrizan, Suzanne MacMahon, Lina Yuan, Rosalind Way, Helena Harder, Rochelle Gold, Amy Taylor, Rhian Gabe, Anneke Lucassen, Ranjit Manchanda, Lesley Fallowfield, Valerie Jenkins, Ashu Gandhi, D. Gareth Evans, Angela George, Michael Hubank, Zoe Kemp, Stephen Bremner, Clare Turnbull

**Affiliations:** 1 Institute of Cancer Research, Division of Genetics and Epidemiology, Sutton, UK; 2 Brighton and Sussex Clinical Trials Unit, Brighton and Sussex Medical School, Brighton, UK; 3 Department of Clinical Genetics, St Georges Hospital NHS Trust, London, UK; 4 Clinical Genomics Department, Centre for Molecular Pathology, NIHR Cancer Biomedical Research Centre, Royal Marsden NHS Foundation Trust, Sutton, UK; 5 Sussex Health Outcomes Research and Education in Cancer (SHORE-C), Brighton and Sussex Medical School, Brighton, UK; 6 BRCA Journey, Patient Representative, Leeds, UK; 7 Clinical Genetics, East Anglian Medical Genetics Service, Cambridge, UK; 8 Wolfson Institute of Population Health, Queen Mary University of London, London, UK; 9 Nuffield Department of Medicine, University of Oxford, Oxford, UK; 10 Department of Gynaecological Oncology, Barts Health NHS Trust, London, UK; 11 Department of Health Services Research, Faculty of Public Health & Policy, London School of Hygiene and Tropical Medicine, London, UK; 12 School of Cancer Sciences, Faculty of Biology, Medicine and Health, University of Manchester, Manchester Academic Health Science Centre, Manchester, UK; 13 Prevent Breast Cancer Centre, Wythenshawe Hospital Manchester Universities Foundation Trust, Manchester, UK; 14 Nightingale and Genesis Breast Cancer Centre, Manchester University Hospitals NHS Foundation Trust, Manchester, UK; 15 Division of Evolution, Infection, and Genomic Sciences, The University of Manchester, Manchester, UK; 16 Cancer Genetics Unit, Royal Marsden NHS Foundation Trust, London, UK; 17 Breast Oncology Unit, Royal Marsden NHS Foundation Trust, London, UK

**Keywords:** Genetic testing, Breast cancer, Genetic counselling

## Abstract

**Background:**

Genetic testing to identify germline high-risk pathogenic variants in breast cancer susceptibility genes is increasingly part of the breast cancer diagnostic pathway. Novel patient-centred pathways may offer opportunity to expand capacity and reduce turnaround time.

**Methods:**

We recruited 1140 women with unselected breast cancer to undergo germline genetic testing through the BRCA-DIRECT pathway (which includes a digital platform, postal saliva sampling and a genetic counsellor telephone helpline). Ahead of consenting to the test, participants were randomised to receive information about genetic testing digitally (569/1140, 49.9%) or via a pre-test genetic counselling consultation (571/1140, 50.1%).

**Results:**

1001 (87.8%) participants progressed to receive their pre-test information and consented to testing. The primary outcome, uptake of genetic testing, was higher amongst participants randomised to receive digital information compared with those randomised to a pre-test genetic counselling consultation (90.8% (95% CI: 88.5% to 93.1%) vs 84.7% (95% CI: 81.8% to 87.6%), *p* = 0.002, adjusted for participant age and site). Non-inferiority was observed in relation to patient knowledge, anxiety, and satisfaction.

**Conclusions:**

Findings demonstrate that standardised, digital information offers a non-inferior alternative to conventional genetic counselling, and an end-to-end patient-centred, digital pathway (supported by genetic counselling hotline) could feasibly be implemented into breast oncology settings.

**Clinical trial registration:**

The study is registered with, and protocol available on, ClinicalTrials.gov (NCT04842799).

## Introduction

Pathogenic variants (PVs) in genes such as *BRCA1*, *BRCA2* and *PALB2* (BRCA-genes) are associated with elevated risk of breast and other cancers (in particular, ovarian cancer), with well-evidenced interventions for early detection and prevention of disease [1–3]. Historically, germline genetic testing for the BRCA-genes and other cancer susceptibility genes (CSGs) was limited by requirement for laborious fragment-by-fragment gene analysis, consequent high costs and slow turnaround times, and was typically only initiated following referral to clinical genetics of families with a strong family history of relevant cancers. Next Generation Sequencing has transformed laboratory workflows, thus dramatically improving capacity and turnaround time. However, other elements of the clinical-laboratory pathway remain laborious, meaning complex eligibility criteria remain necessary to restrict the patient volume eligible for testing [4, 5].

Women identified at time of breast cancer (BC) diagnosis as carrying a high-risk PV in a BRCA-gene may elect to reduce their risk by having bilateral mastectomy instead of, or following on from, localised surgery [6]. Furthermore, germline BRCA-gene status has emerged as an important therapeutic biomarker in BC informing oncological therapies [7–9]. For example, PARP-inhibitors have recently been approved in the UK by the National Institute of Clinical Excellence (NICE) for HER2-negative advanced (Talazoparib) and early-stage (Olaparib) BC with germline BRCA PVs [10, 11]. Consequently, BRCA-gene testing is increasingly offered to BC patients contemporaneous to diagnosis to inform treatment options.

Due to a lack of capacity in clinical genetics and the delay inherent in referral, there has been increasing momentum for delivery for diagnostic genetic testing in the ‘mainstream’ oncology setting [12]. Implementation of this has however had variable success and acceptance owing to (i) perceived lack of expertise regarding genetic information-giving by the mainstream clinicians/surgeons themselves, (ii) lack of time in oncology appointments for detailed information-giving, counselling and consenting, (iii) perception of this role being outside the oncology remit, and (iv) navigation of complex eligibility criteria [13].

We hypothesised that most elements of the germline genetic testing process for BC patients were generic and amenable to a more standardised delivery process. We hypothesised that, if required, complex psychological, legal, clinical and/or risk-based information provision and counselling could be provided in a responsive patient-centred model and there would be benefit to this being available throughout the testing process, rather than at just one fixed timepoint. Such a pathway could minimise the patient-facing time and administrative burden to oncology professionals for delivery of BRCA-testing. We therefore designed a digital pathway termed BRCA-DIRECT, comprising (i) an end-to-end online digital workflow portal providing all generic elements of the testing process (including delivery of information about genetic testing (or ‘pre-test information’), documentation of consent, and return of negative results), (ii) postal saliva sampling, and (iii) a genetic counsellor hotline. We conducted the BRCA-DIRECT pathway study in unselected BC patients across breast oncology units in UK hospitals between 2021 and 2023. The study incorporated a randomised non-inferiority comparison of pre-test information delivery via the digital platform versus via a genetic counselling telephone consult.

## Methods

### Patients

#### Recruitment

Patients were invited to participate in BRCA-DIRECT by clinical or research teams from five breast oncology units within two National Health Service (NHS) trusts in Manchester or London, UK.

A two-stage consent process was required to initiate genetic testing.

Firstly, the patient was provided with information about the BRCA-DIRECT study within clinic. Patients could ‘express interest’ in participating or provide a reason for decline. Those expressing an interest were given a study pack (including research consent form and a saliva sampling kit) to complete at home and return by post.

**Fig. 1:**
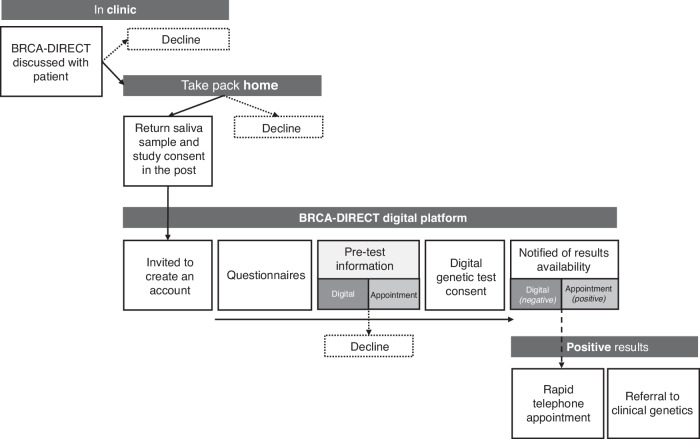
The BRCA-DIRECT Pathway. Flow diagram of steps completed in clinic, at home and online via the BRCA-DIRECT website by participants  during the BRCA-DIRECT testing pathway.

Secondly, after signing the study consent, participants were invited via email and/or SMS to create an account on the BRCA-DIRECT website, which gave them access to a personalised dashboard, providing an overview of the steps involved in the genetic testing (GT) pathway. The platform automatically notified and enabled completion of time-stamped tasks (see previously published description and Fig. 1) [14]. This included a digital genetic test consent form, only available after receiving the pre-test information (see randomisation).

A telephone genetic counselling hotline was available between 9 am and 5 pm, Monday-to-Friday, through which a genetic counsellor/genetic nurse (GC/GN) could provide support for clinical enquiries, as well as for administrative or technical help.

#### Participant eligibility

The following criteria were applied:

- Inclusion:
  - Diagnosis of invasive BC or high-grade ductal carcinoma in-situ (DCIS);
  - Female;
  - Over the age of 18 years old;
  - Good comprehension of English language; and
  - Access to internet with an email and/or telephone number (this could be via a trusted friend or family-member).
- Exclusion:
  - Previous BRCA-gene testing.

#### Participant demographics

Participant demographics were collected via a digital survey at baseline. Information relating to BC type and status (newly diagnosed, in follow-up, or metastatic) was collected by local hospital teams at point of consent. Date of primary BC surgery, where available, was recorded prior to study closure.

### Genetic testing

GT was conducted within an NHS accredited diagnostic laboratory at the Royal Marsden NHS Foundation Trust. All samples from participants consenting to GT underwent full gene sequencing of *BRCA1*, *BRCA2* and *PALB2*, including copy number variant analysis. Variants were reported if classified as pathogenic, likely pathogenic, or ‘hot’ variant of uncertain significance (VUS), namely those scoring just under the threshold for likely pathogenic (with 5 evidence points).

### Randomisation

#### Pre-test information randomisation

Participants were randomised 1:1 to receive pre-test information digitally via the BRCA-DIRECT website (fully-digital arm) or via a telephone pre-test genetic counselling consultation with a GN/GC (partially-digital arm). Allocation to arm was based on pre-generated, site-specific randomisation lists from the on-line Sealed Envelope randomisation list generator [15]. IDs were allocated to participants sequentially as they were registered to BRCA-DIRECT. Participants were only aware of their allocation after they had completed the baseline questionnaires and had proceeded to the point of receiving the pre-test information.

Digital pre-test information consisted of 21 static screens of text and schematics designed to be equivalent in detail and depth to a standard genetic counselling appointment.

Those allocated to receive the pre-test genetic counselling consultation were invited to book an appointment within the next 3-working days (rolling basis). Participants who did not answer the telephone within the slot were notified to rebook an appointment online.

#### Results randomisation

Participants were also pre-allocated to receive results digitally (97.5%) or via a telephone appointment with a GN/GC (2.5%). This randomisation was over-ridden if the participant (i) had a positive genetic test result and (ii) had been randomised to receive their result digitally. All participants with positive results, in addition to those randomised to this arm, were issued an online invitation to book a telephone consultation with a GN/GC.

### Study outcomes

#### Non-inferiority of digital pre-test information

The main study aim was to evaluate non-inferiority of digital pre-test information compared with a pre-test genetic counselling consultation.

The primary outcome was uptake of GT. The following secondary outcomes were also evaluated: patient-reported anxiety (State Trait Anxiety Index and Intolerance of Uncertainty); knowledge about genetic testing (14-point study specific questionnaire); and satisfaction with pre-test information delivery (measured on a five-point Likert Scale, within the patient satisfaction survey) [16, 17]. More detail on the methods and timepoints for assessment are presented in supplementary table 1, with assumptions used to establish the non-inferiority margins presented in supplementary table 2.

### Feasibility and acceptability outcomes

In addition to non-inferiority outcomes, we aimed to understand broader feasibility and acceptability of the pathway by measuring:

- Overall uptake of the digital pathway, based on expressions of interest and progression to receive pre-test information.
- Uptake of the genetic counselling hotline by consented participants, based on call logs.
- Healthcare professional (HCP) satisfaction with the BRCA-DIRECT pathway. A digital survey was circulated to breast oncology, surgical and clinical genetics HCPs from the recruiting sites between August and September 2022 to understand perception of how elements of the pathway compared to current standard-of-care and views on suitability of the pathway for broader rollout.

We also conducted structured interviews with participants to explore the motivations for, and experiences of, BRCA-testing via the BRCA-DIRECT digital pathway at an early stage in their breast cancer diagnosis and treatment. Findings from these interviews will be reported separately.

### Statistical methods and analyses

#### Sample size

Study sample size (1000 participants) was calculated to ensure >95% likelihood of identifying at least five individuals with a pathogenic variant at each of the two recruiting trusts, based on a PV-detection rate of 2%. The sample sizes required to achieve 80% power for individual non-inferiority outcomes are presented in supplementary table 1.

#### Analyses

The primary outcome was analysed using a logistic regression model, with fixed effects for randomisation arm and site. Secondary non-inferiority outcomes were analysed using linear mixed effects models with random effect for participant and fixed effects for time point, baseline measure of outcome, randomisation arm and site. Age was added as a covariate to all models a priori to account for the known associations with age and digital accessibility. Baseline outcome measures were included where assessed. Trait anxiety and intolerance of uncertainty scores were included within the model for anxiety.

Sensitivity analyses were performed to identify effects of completing outcome measures outside of time window. Sub-group analyses were performed, where possible, considering the following parameters: localised/advanced BC; genetic test result; reported family history (BC and other cancers <40 years old in first- or second-degree relatives); and method of receiving result.

Analyses were completed following intention-to-treat principles in Stata v17.0.

## Results

### Uptake of digital pathway study for genetic testing

**Fig. 2:**
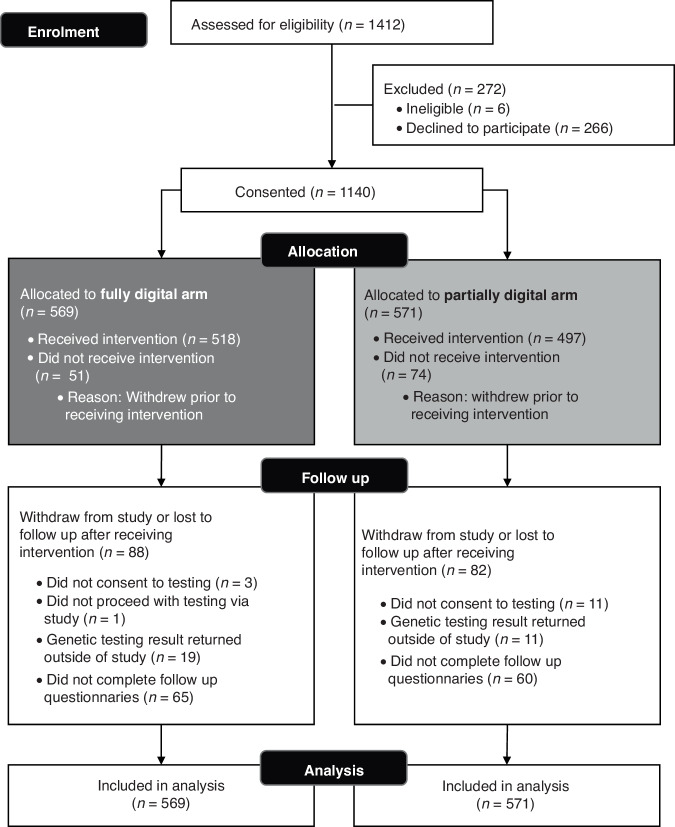
CONSORT flow chart for recruitment to the BRCA-DIRECT study. Participants split depending on allocation to the fully digital arm (digital pre-test information) or the partially digital arm (pre-test genetic counsellor consultation) following consent to study.

During the recruitment period (05/07/2021 to 15/08/2022 (406 days)), 1412 patients expressed interest and were provided with a study pack (see CONSORT Fig. 2). Limited information was collected regarding upfront decline from the London sites. However, of those approached in Manchester, excluding those ineligible on account of having already had testing, 248/872 (28.4%) people declined upfront, with the main reasons being: no internet access (93/248, 37.5%), not interested in study participation (67/248, 27.0%), and not interested in BRCA-testing (48/248, 19.4%).

Of the 1412 who expressed interest upfront, 1140 (80.7%) patients returned their study consent and saliva sample in the post within the recruitment window, with 569 (49.9%) allocated to the fully-digital arm and 571 (50.1%) allocated to the partially-digital arm.

Final follow-up of all participants was completed on 16/01/2023.

### Participant characteristics

The mean age (±SD) of participants was 58.6 (±11.7) years. A majority of participants were white (84.1%), married or partnered (68.4%), and in full or part-time work (57.3%) with 49.9% of participants educated to degree level or higher (see Table 1).

**Table 1:** Participant demographics

	Overall (*n* = 1140)					
	Partially digital (*n* = 571)		Fully digital (*n* = 569)		Overall (*n* = 1140)	
	Mean	±SD	Mean	±SD	Mean	±SD
**Age**	58.1	11.6	59.2	11.77	58.6	11.72
	*n*	%	*n*	%	*n*	%
**Age group**						
18–30 years old	0	0	1	0.2	1	0.1
31–40 years old	31	5.4	26	4.6	57	5
41–50 years old	130	22.8	113	19.9	243	21.3
51–60 years old	177	31	176	30.9	353	31
61–70 years old	146	25.6	149	26.2	295	25.9
71–80 years old	66	11.6	89	15.6	155	13.6
81+ years old	21	3.7	15	2.6	36	3.2
*Total with available data*	571	100	569	100	1140	100
*Missing/prefer not to say*	0	0	0	0	0	0
**Ethnicity**						
White	418	80.5	466	87.6	884	84.1
Asian or Asian British	41	7.9	26	4.9	67	6.4
Black African/Caribbean or Black British	20	3.9	15	2.8	35	3.3
Mixed or multiple ethnicities	14	2.7	14	2.6	28	2.7
Other	26	5	11	2.1	37	3.5
*Total with available data*	519	90.9	532	93.5	1051	92.2
*Missing/prefer not to say*	52	9.1	37	6.5	89	7.8
**Highest qualification**						
No qualifications	31	6.2	40	7.7	71	7
GCSE or equivalent	116	23.2	105	20.3	221	21.7
NVQ or equivalent	73	14.6	60	11.6	133	13.1
A Levels	35	7	50	9.7	85	8.3
Degree	165	32.9	165	31.9	330	32.4
Higher degree	81	16.2	97	18.8	178	17.5
*Total with available data*	501	87.7	517	90.9	1018	89.3
*Missing/prefer not to say*	70	12.3	52	9.1	122	10.7
**Marriage status**						
Single	68	13.2	64	12.2	132	12.7
Married or partnered	349	67.9	362	68.8	711	68.4
Divorced	60	11.7	53	10.1	113	10.9
Widowed	37	7.2	47	8.9	84	8.1
*Total with available data*	514	90	526	92.4	1040	91.2
*Missing/prefer not to say*	57	10	43	7.6	100	8.8
**Employment status**						
Unemployed	34	6.8	38	7.3	72	7.1
Working part time	111	22.3	111	21.4	222	21.8
Working full time	188	37.8	173	33.3	361	35.5
Retired	165	33.1	197	38	362	35.6
*Total with available data*	498	87.2	519	91.2	1017	89.2
*Missing/prefer not to say*	73	12.8	50	8.8	123	10.8
**Breast cancer status**						
New diagnosis, pre-surgical	155	27.2	155	27.2	310	27.2
New diagnosis, pre-surgical, neoadjuvant, chemo	75	13.2	59	10.4	134	11.8
New diagnosis, post surgical	120	21.1	103	18.1	223	19.6
In follow-up	188	33	212	37.3	400	35.1
Metastatic breast cancer	31	5.4	40	7	71	6.2
*Total with available data*	569	99.6	569	100	1138	99.8
*Missing*	2	0.4	0	0	2	0.2
**Reported family history of cancer**						
Yes - Family history reported	232	44.6	241	44.8	473	44.7
No family history reported	288	55.4	297	55.2	585	55.3
*Total with available data*	520	91.1	538	94.6	1058	92.8
*Missing*	51	8.9	31	5.4	82	7.2

### Non-inferiority

#### Consent to and uptake of genetic test

Out of 1140 participants, 1001 (87.8%) proceeded to consent to GT (515/569 in the fully-digital arm and 486/571 in the partially-digital arm), whilst 139 (12.2%) did not (54/569 in the fully-digital arm and 85/571 in the partially-digital arm).The adjusted proportions of uptake in each arm were 84.7% (95% CI 81.8% to 87.6%) uptake in the partially-digital arm and 90.8% (88.5% to 93.1%) in the fully-digital arm (*p* = 0.002). The adjusted difference between the arms was +6.1% (2.4% to 9.8%) (supplementary figure 1a, Table 2). Therefore, uptake of genetic testing in the fully-digital arm was non-inferior (and also superior) to the partially-digital arm.

**Table 2:** Non-inferiority outcomes

	Observations (participants)	Fully-digital (digital pre-test information), percentage or mean (95% CI)	Partially-digital (pre-test genetic counselling consultation), percentage or mean (95% CI)	Difference (95% CI) *P*-value	non-inferiority margin and conclusion
Uptake of Genetic Testing	1140 (1140)	90.8% (88.5%–93.1%)	84.7% (81.8%–87.6%)	6.1% (2.4% to 9.8%) *p* = 0.002	5.5% non-inferior
Anxiety	2753 (994)	38.3 (37.7–39.0)	37.8 (37.1–38.5)	0.5 (−0.4 to 1.4) *p* = 0.269	+3.0 non-inferior
Knowledge	1755 (989)	8.2 (7.9–8.4)	9.2 (9.0–9.5)	−1.1 (−1.4 to -0.8) *p* < 0.001	−1.4 non-inferior
Participant satisfaction	908 (908)	4.7 (4.6–4.7)	4.7 (4.6–4.7)	−0.002 (−0.1 to 0.1) *p* = 0.962	−0.75 non-inferior

Included within the group who did not consent to GT were participants who failed to register on the platform (51.1%, *n* = 71), did not proceed with the digital baseline activities (38.8%, *n* = 54), or withdrew after receiving the pre-test information (10.1%, *n* = 14).

#### Patient knowledge about genetic testing

Knowledge scores ranged from 0 to 14, representing the proportion of answers scored correctly within the knowledge questionnaire. Knowledge scores increased from baseline in both arms, at both timepoints measured (1-day post genetic test consent and 28-days post results) (supplementary figure 2). Overall adjusted mean knowledge scores were 9.22 (95% CI: 9.00–9.45) in the partially-digital arm and 8.15 (95% CI: 7.93–8.37) in the fully-digital arm. The adjusted effect of arm on knowledge score was −1.07 (95% CI: −1.39 to −0.75) in the fully-digital arm compared to the partially-digital arm (*p* < 0.001) (Supplementary Fig. 1b, Table 2). Based on the non-inferiority (NI) margin (−1.40), the fully-digital arm was non-inferior to the partially-digital arm in relation to knowledge about GT.

#### Patient anxiety

State anxiety scores, as measured by the State Trait Anxiety Index, ranged from 20 to 80, with a lower score representing less anxiety. Overall adjusted mean anxiety scores were 37.79 (95% CI: 37.13–38.46) in the partially-digital arm and 38.31 (95% CI: 37.66 to 38.96) in the fully-digital arm, with anxiety decreasing over time from baseline (supplementary figure 3). The adjusted effect of arm on anxiety score was 0.51 points (95% CI: −0.41 to 1.44) in the fully-digital arm compared to the partially-digital arm (*p* = 0.277) (supplementary figure 1c, Table 2). The NI margin was +3, therefore, the fully-digital arm was non-inferior to the partially-digital arm in terms of reported anxiety.

#### Patient Satisfaction

In both arms, >90% of participants reported satisfaction scores of four or five, out of five, on the Likert scale (1- very unsatisfied; 5 – very satisfied). Adjusted mean satisfaction scores were 4.67 (95% CI: 4.61–4.73) in the partially-digital arm and 4.67 (95% CI: 4.60–4.73) in the fully-digital arm. The adjusted effect of arm on patient satisfaction score was −0.002 points on Likert scale (95% CI: −0.09 to 0.09) in the fully-digital arm compared to the partially-digital arm (*p* = 0.962) (supplementary figure 1d, Table 2), thus the fully-digital arm was non-inferior to the partially-digital arm in terms of patient satisfaction.

#### Subgroup and sensitivity analyses

There were no effects observed on the outcomes of non-inferiority analyses following sensitivity or subgroup analyses (see supplementary table 3a–d).

### Genetic testing results

Of those who consented to GT, two participants subsequently withdrew from testing via the study. The overall pick-up rate of PVs was 3.0% (30/999; 10/30 *BRCA1*, 12/30 *BRCA2*, and 8/30 *PALB2)*. No variants of uncertain significance were reported.

Overall, 57/999 (5.7%) results were returned via a consultation with a GC (including, all 30 participants with a PV result and 27 participants with a negative result who were pre-allocated to the consultation arm). The remainder of results (942/999, 94.3%) were returned digitally according to pre-allocated randomisation.

### Test-offer-to-results-time

Median (interquartile range (IQR)) turnaround time of results was 54.0 (24.0–69.0) days for Manchester and 47.0 (36.0–63.5) days for London, and similar between the two arms (partially-digital: 49.0 (37.0–67.0) days; fully-digital 49.0 (39.0–66.0) days).

667/1140, or 58.5%, of participants were newly diagnosed with a BC at point of recruitment and of these, 407/667 (or 65.6%) had not yet undergone primary surgical treatment for their BC. 378/407 (92.9%) subsequently proceeded to consent to genetic testing and of these 222/378 (58.7%) received their result before their planned surgery date (where this was known).

### Hotline utilisation

Hotline call logs recorded by the study team covered the period 21/07/2021 to 11/01/2023. During which time, calls were recorded from 201/1140 (17.6%) participants: 90/569 (15.8%) of whom had been allocated to the fully-digital arm and 111/571(19.5%) of whom had been allocated to the partially-digital arm. Overall, 324 hotline call logs were recorded (amounting to 1,441 minutes of calls) of which 50 (15.4%) were clinical and 274 (84.6%) were administrative.

### Healthcare professional satisfaction

Responses were recorded from 37 healthcare professionals (16 Manchester and 21 London). 19/37 (51.4%) respondents were consultant breast oncologists or surgeons with other respondents comprising oncology clinical nurse specialists, research nurses, genetic counsellors, clinical geneticists, and trainee oncologist/surgeons.

**Fig. 3:**
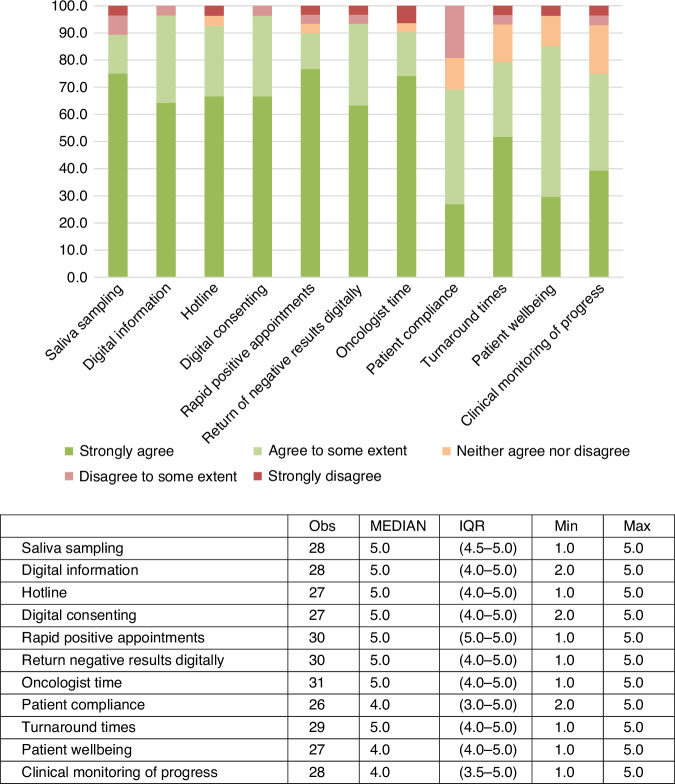
Healthcare professional satisfaction with the BRCA-DIRECT pathway. Healthcare professional reported agreement (1 strongly disagree, 5 – strongly agree), on whether aspects of the BRCA-DIRECT pathway were equivalent (or superior) in comparison to current standard-of-care.

On average, healthcare professionals expressed agreement (either 4 (agree to some extent) or 5 (strongly agree)) that all aspects of the pathway were equivalent, or superior, to current standard-of-care, with an overall median score (IQR) of 4.5 (4.3 to 5.0) factoring in all aspects of the pathway (Fig. 3). The lowest scores were recorded for consideration against current standard-of-care in regard of ‘patient compliance with the pathway’ (4.0 (3.0 to 5.0)) and ‘clinical monitoring of patient progress’ (4.0 (3.5–5.0)).

## Discussion

To our knowledge, this is the largest study reported to date of germline genetic testing using a digital pathway in cancer patients. We have demonstrated logistical feasibility and acceptability of a patient-centred, digital pathway for mainstreaming of BRCA-testing for unselected female BC patients. Via randomised comparison, we also showed non-inferiority against a range of outcomes of digital delivery of pre-test information compared with pre-test genetic counselling consultation.

We observed attrition of participants upstream of (i) consenting to the research study and (ii) receiving the pre-test information (in total, 19.3% of those initially expressing interest, Fig. 2). Because of the nature of the pathway, there was limited opportunities for collection of data regarding non-progression, however, based on the limited evidence gathered from Manchester with regards reasons for upfront decline, it could be surmised that at both stages, attrition may have partly been a consequence of the research study around the BRCA-testing pathway, rather than relating to the BRCA-testing pathway itself. For example, there was need for both research consent as well as standard genetic testing consent, which in some cases proved confusing to patients. Furthermore, being a research study placed additional time requirements on participants for completion of questionnaires. However, this attrition may also reflect barriers (for example, lack of internet access) to inclusion within the study, progression through the pathway, or willingness to participate in GT.

The demographics of participating patients are broadly reflective of BC patients nationally, albeit slightly younger in average age of diagnosis (59 compared with 62 years old). However, there plausibly may be socio-demographic differences in characteristics between all patients presenting in clinic for BC management during the study window and those taking up BRCA-testing. These may reflect biases relating to those offered the study, those expressing interest following offer, and those progressing digitally to the point of test uptake (genetic test consent). Such factors are relevant to the generalisability of the findings; similar factors may well pertain to uptake of BRCA-testing through existing standard pathways and warrant further exploration to improve equity of access to BRCA-testing more generally. However, these biases should not be differential between the two arms of the study, and as such, for those who do opt for testing via a digital pathway, the observation of superior test uptake in the fully-digital arm is of note.

Indeed across all outcomes, our randomised comparison demonstrated non-inferiority of digital delivery of pre-test information compared with a pre-test genetic counselling consultation. Though it is important to consider that our comparator arm was a “partially-digital” pathway, limited to comparison of just the pre-test information, rather than a true standard-of-care arm for BRCA-testing. Nonetheless, our findings relating to equivalence of patient-reported anxiety and satisfaction were promising. Additionally, the relatively low demand for clinical support via the genetic counselling hotline similarly indicate low proportions with substantial anxiety or need for extra support throughout the process.

The genetic counselling hotline was intended to provide optional access to a GN/GC to supplement digital pre-test information. Thus, patient contact with a GN/GC is not necessitated within the proposed pathway but rather allows patient-centred access to support if required. This differs from previous studies in cancer patients, which have largely focussed on implementing digital information or tools in addition (or prior) to genetic counselling [18, 19].

Two exceptions to this, with similar findings, are a US study, reported by Swisher et al., that reported noninferiority of patient distress at three months and no statistically significant differences in anxiety, depression, or decisional regret between participants who did not have mandatory pre-test counselling (but instead received information in the format of a video) compared to those who had pre-test genetic counselling [20]. However, this study was not delivered in a mainstream oncology setting for patients under active management for a breast cancer, but recruited participants from the public with a personal or family history of breast or ovarian cancer.

Another study reported by Sie et al. (2013), gave 161 selected BC patients referred to clinical genetics services within the Netherlands the option to proceed with testing via a fully digital route, as alternative to conventional consultation (there was no genetic counselling hotline in this study). Sie et al. also reported high uptake in the digital pathway, that more patients preferred testing without prior face-to-face counselling, and similar outcomes in relation to distress (anxiety) and satisfaction between the digital arm and conventional clinical genetics consultation arm [21].

Within our study, we also compared knowledge between the two arms and our findings reveal that, whilst lower, mean patient knowledge scores in the fully-digital arm lay within the pre-set margin of non-inferiority. Therefore, the standardised material we produced with our patient involvement group can be deemed suitable and sufficient for providing pre-test information generating acceptable knowledge levels, as compared to a GC/GN consult. However, a priority area for future exploration is improving inclusivity and accessibility of digital materials to meet diverse patient needs and to ensure that we are not exacerbating health inequalities. For example, for different learning styles, reading levels, or languages, and accessible content for those with hearing or sight loss, which could be achieved via more interactive tools, use of audio-visual content, or physical paper-based information as an adjunct.

Previous studies of digital pathways have largely focused on community-based ascertainment for genetic testing, for example BRCA founder mutation testing for those with Jewish ancestry. Our study is the first focussed on using a digital pathway for patients under active oncology management within the UK NHS. At both sites involved in the study, HCP satisfaction with the pathway was overall high, with expression of readiness for broader rollout. Our clinician satisfaction data may reflect bias regarding those clinicians electing to participate in the survey; furthermore, the two trusts involved in the study already had well-established mainstreaming pathways for BC GT and thus their clinician population may not be reflective nationally. Improvements in HCP feedback relating to turnaround times were observed compared to the pilot study, likely reflecting improvements in laboratory test turnaround times (which improved over the study period following COVID19-related laboratory delays and optimisation of copy number variant analysis for saliva-derived DNA).

In terms of the patient population, we offered GT to all women attending for BC diagnosis, management, or follow-up, for which the observed pick up rate from GT (3.0%) was in line with other studies of testing in unselected BC. UK health economic analysis undertaken in 2018 suggest universal testing of *BRCA1, BRCA2* and *PALB2* genes for BC patients to be economically impactful within NICE willingness to pay thresholds of £30,000/QALY up to a per-patient cost of £1,626 (payer perspective) and £1,868 (societal perspective) [22]. Of note, recent guidance from the American Society of Clinical Oncology (ASCO) has recommended testing for *BRCA1* and *BRCA2* in all BC cases up to the age of 65, including those diagnosed historically [23]. Analyses including the patients recruited through Manchester centres have identified that fewer than 20% of women with unselected BC included in this study would be eligible for BRCA-testing under current NHS National Test Directory criteria, with approximately half of the total *BRCA1/BRCA2/PALB2* pathogenic variants occurring in this ineligible group [24]. This analysis demonstrated that detection of PVs is higher in cases with younger onset, higher-grade, bilateral and/or hormone-receptor negative disease, and where a relevant family history is present [24]. There is, therefore, an inherent tension in balancing enhancement of detection rate against the complexity and imperfect sensitivity incurred from imposing complex test eligibility criteria.

This study, as well as the aforementioned health economics study, focused on multigene panel testing of *BRCA1, BRCA2 and PALB2*, reflecting the NHS National Test Directory (NTD) ‘R208’ indication at the start of recruitment. This indication has since been extended to include an additional four, lower penetrance genes (*ATM, CHEK2, RAD51C*, and *RAD51D*). Thus, there is now additional complexity regarding potential expansion of eligibility criteria, with consideration also of the impact of identifying more PVs (and VUSs) in lower penetrance genes for which there is less clinical actionability (regarding both breast cancer oncological therapy and screening/prevention of future cancers). More research is, therefore, required to understand the capacity pressures and health economic impact of such expansion, as well as the impact on both clinicians and patients receiving and managing these results.

Nevertheless, any substantial expansion in BRCA-testing of BC cases, for example testing of all cases arising age ≤65 years, would require higher-throughput clinical and laboratory systems. We propose that such systems should (i) incorporate more generic patient information materials and clinical workflows to enable scale, (ii) retain ready access to expert clinical input individualised to patient requirements and (iii) attune flexibly to local clinical pathways and informatic workflows, especially as GT timed at point of diagnosis requires efficient timely delivery. Additional work is required to understand necessary facilitators and adaptations to optimise equity of access; pathway-specific health economic analyses will also be important.

Overall, this study demonstrates that, supported by a genetic counselling hotline, a fully-digital pathway may be a suitable alternative to conventional models of pre-test information-giving, sampling and consenting, and could support end-to-end management of genetic testing for a large proportion of BC patients. Where expansion of germline genetic testing is limited by clinical capacity, pathways such as BRCA-DIRECT, implemented within mainstream oncology clinics may offer a viable and acceptable approach for the majority of patients to minimise the patient-facing and administrative burdens to clinicians of BRCA-testing, whilst providing flexible patient access to clinical genetics expertise. Thus enabling both clinical genetics and oncology professional time to be utilised more effectively for management of those with a positive result or supporting a minority of patients for whom a standardised, digital approach is unsuitable.

## Supplementary information

Supplementary Material

## Data Availability

The datasets generated and/or analysed during the current study are available from the corresponding author on reasonable request.
