# Supplementary material for: BRCA-DIRECT digital pathway for diagnostic germline genetic testing within a UK breast oncology setting: a randomised, non-inferiority trial
Source: Br J Cancer. 2024 Oct 1;131(9):1506–15. doi: 10.1038/s41416-024-02832-2 (PMC11519354; doi:10.1038/s41416-024-02832-2)
Supplement: Supplementary file 1 — Supplementary Material [file 41416_2024_2832_MOESM1_ESM.docx]

**Supplementary materials**

**Title:**

BRCA-DIRECT digital pathway for diagnostic germline genetic testing within a UK breast oncology setting: a randomised, non-inferiority trial

**Authors:**

Bethany Torr^1^, Christopher Jones^2^, Grace Kavanaugh^1^, Monica Hamill^1^, Sophie Allen^1^, Subin Choi^1^, Alice Garrett^1,3^, Mikel Valganon-Petrizan^4^, Suzanne MacMahon^4^, Lina Yuan^4^, Rosalind Way^1^, Helena Harder^5^, Rochelle Gold^6^, Amy Taylor^7^, Rhian Gabe^8^, Anneke Lucassen^9^, Ranjit Manchanda^8,10,11^, Lesley Fallowfield^5^, Valerie Jenkins^5^, Ashu Gandhi^12,13^, D. Gareth Evans^14,15^, Angela George^1,16^, Michael Hubank^4^, Zoe Kemp^1,16,17^, Stephen Bremner^2^, and Clare Turnbull^1,16^

**Affiliations**

1. Institute of Cancer Research, Division of Genetics and Epidemiology, Sutton, UK
2. Brighton and Sussex Clinical Trials Unit, Brighton and Sussex Medical School, Brighton, UK
3. Department of Clinical Genetics, St Georges Hospital NHS Trust, London, UK
4. Clinical Genomics Department, Centre for Molecular Pathology, NIHR Cancer Biomedical Research Centre, Royal Marsden NHS Foundation Trust, Sutton, UK
5. Sussex Health Outcomes Research and Education in Cancer (SHORE-C), Brighton and Sussex Medical School, Brighton, UK
6. BRCA Journey, Patient Representative, Leeds, UK
7. Clinical Genetics, East Anglian Medical Genetics Service, Cambridge, UK
8. Wolfson Institute of Population Health, Queen Mary’s University of London, London, UK
9. Nuffield Department of Medicine, University of Oxford, Oxford, UK
10. Department of Gynaecological Oncology, Barts Health NHS Trust, London, UK
11. Department of Health Services Research, Faculty of Public Health & Policy, London School of Hygiene and Tropical Medicine, London, UK
12. School of Cancer Sciences, Faculty of Biology, Medicine and Health, University of Manchester, Manchester Academic Health Science Centre, Manchester, UK
13. Prevent Breast Cancer Centre, Wythenshawe Hospital Manchester Universities Foundation Trust, Manchester, UK
14. Nightingale and Genesis Breast Cancer Centre, Manchester University Hospitals NHS Foundation Trust, Manchester, UK
15. Division of Evolution, Infection, and Genomic Sciences, The University of Manchester, Manchester, UK
16. Cancer Genetics Unit, Royal Marsden NHS Foundation Trust, London, UK
17. Breast Oncology Unit, Royal Marsden NHS Foundation Trust, London, UK

**Corresponding author**:

Prof Clare Turnbull, Division of Genetics and Epidemiology, Institute of Cancer Research, London SM2 5NG, UK; [clare.turnbull@icr.ac.uk](mailto:clare.turnbull@icr.ac.uk); <https://orcid.org/0000-0002-1734-5772>

Contents

[Supplementary Figure 1: Adjusted differences between the fully-digital arm compared to the partially-digital arm. 3](#_Toc173329231)

[(a) uptake of genetic testing 3](#_Toc173329232)

[(b) mean knowledge score 3](#_Toc173329233)

[(c) mean anxiety scores 3](#_Toc173329234)

[(d) mean satisfaction scores 3](#_Toc173329235)

[Supplementary Figure 2: Baseline and adjusted mean knowledge scores in the fully- and partially-digital arms over time 4](#_Toc173329236)

[Supplementary Figure 3: Baseline and adjusted mean anxiety scores in the fully- and partially-digital arms over time 4](#_Toc173329237)

[Supplementary Table 1: Methods of evaluating outcomes for assessing non-inferiority of digital information with the timepoints of assessment. 5](#_Toc173329238)

[Supplementary Table 2: Non-inferiority margin assumptions and power 6](#_Toc173329239)

[Supplementary Table 3: Sensitivity and subgroup analyses 7](#_Toc173329240)

[(a) Genetic test uptake 7](#_Toc173329241)

[(b) Knowledge 8](#_Toc173329242)

[(c) Anxiety 9](#_Toc173329243)

[(d) Satisfaction 10](#_Toc173329244)

[Supplementary table 4: Uptake of genetic testing by participant demographics 11](#_Toc173329245)

[References 12](#_Toc173329246)

# Supplementary Figure 1: Adjusted differences between the fully-digital arm compared to the partially-digital arm.

δ Indicates the non-inferiority margin.

## (a) uptake of genetic testing

**
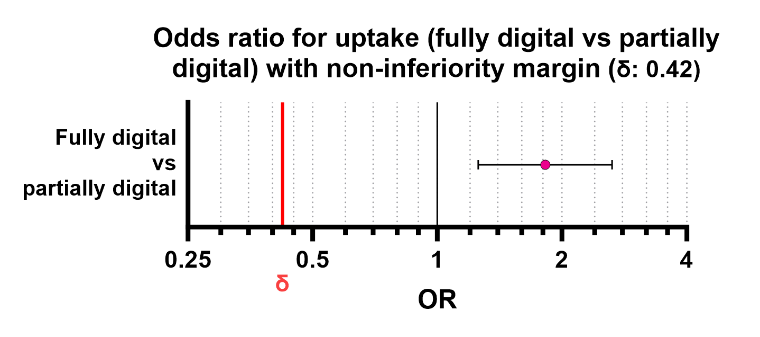
**

OR = Odds ratio

(b) mean knowledge score**
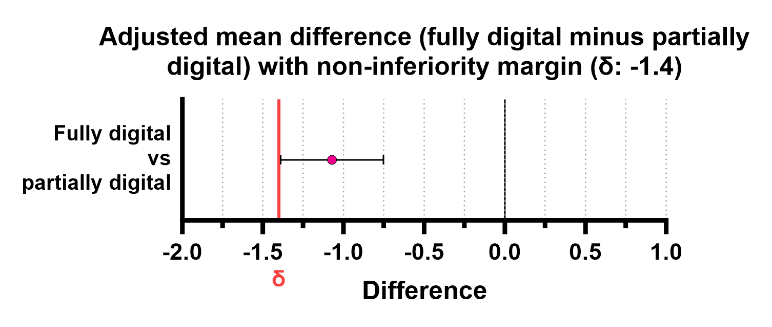
**

## (c) mean anxiety scores

**
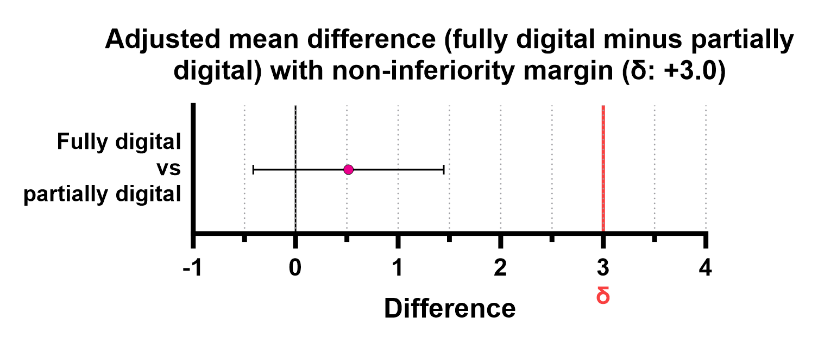
**

(d) mean satisfaction scores
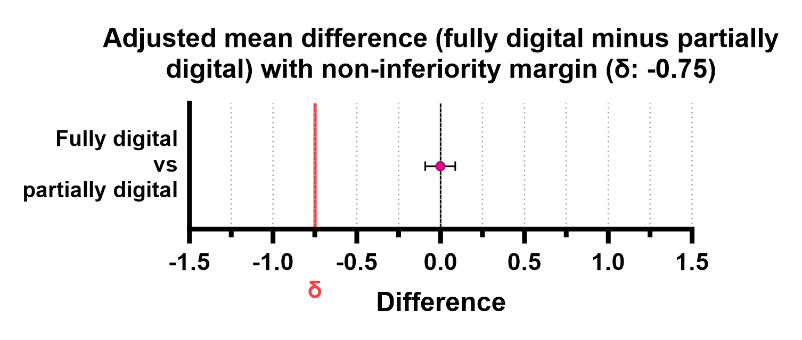


# **Supplementary Figure 2: Baseline and adjusted mean knowledge scores in the fully- and partially-digital arms over time**

# **Supplementary** Figure 3: **Baseline and adjusted mean anxiety scores in the fully- and partially-digital arms over time**

T1 = 1-day post genetic test consent

T2 = 7-days post genetic test results

T3 = 28-days post genetic test results

# Supplementary Table 1: Methods of evaluating outcomes for assessing non-inferiority of digital information with the timepoints of assessment.

| **Outcome** | **Measure** | **Timepoint of assessment** | | | | **Non-inferiority Margin** (sample size required for 80% power) |
| --- | --- | --- | --- | --- | --- | --- |
|  |  | Baseline | 1-day after genetic test consent (T1) | 7-days after receiving the genetic test result (T2) | 28-days after receiving the genetic test result (T3) |  |
| **Genetic test uptake** | Proportion of participants consenting to genetic testing | N/A | | | | -5.5%  (934) |
| **Anxiety** | State Trait Anxiety Inventory  20-item validated survey, producing state and trait scores between 20 and 80, with higher scores reflecting greater anxiety. ^19^ | - Trait and state anxiety | - State anxiety | - State anxiety | - State anxiety | +3  (636) |
|  | Intolerance of uncertainty  ^20^ |  |  |  |  |  |
| **Knowledge** | 14-item study specific questionnaire, with true or false answers. Participants could also answer ‘don’t know’. Scores reflected total correct answers out of a possible 14. |  |  |  |  | -1.4  (56) |
| **Participant satisfaction** | 10-item study specific survey.  Focussed question on satisfaction with the method of receiving pre-test information Scored on a 5-point Likert scale (1- very unsatisfied; 5 – very satisfied). |  |  | (invited at T2 with reminder at T3 if incomplete) | | -0.75  (38) |

# Supplementary Table 2: Non-inferiority margin assumptions and power

|  | **Attrition** | **Predicted available participants (following attrition)** | **Assumptions**  **(reference *)** | **Power** | **Sample size required for 80% power (digital: clinician = 1:1)** | **Significance level (one-sided)** | **Non-inferiority margin** | **Notes** |
| --- | --- | --- | --- | --- | --- | --- | --- | --- |
| **Test uptake** | 0% | 1000 | 90% test uptake in both groups | 80-85% with 1000 participants | 892 | 0.05 | 5% | Calculated using [http://www.hwasoon.kim/NISSC/#!/binary](http://www.hwasoon.kim/NISSC/) |
| **STAI state T1** | 0% | 1000 | Mean 43.2, SD 13.5 ^1^  *(range 0 – 100)* | >95% with 1000 participants | 500 | 0.05 | 3 | Calculated using [http://www.hwasoon.kim/NISSC/#!/continuous](http://www.hwasoon.kim/NISSC/) |
| **STAI state T2** | 10% | 900 | Mean 43.2, SD 13.5 ^1^  (range 0 – 100) | >95% with 900 participants | 500 | 0.05 | 3 |  |
| **STAI state T3** | 20% | 800 | Mean 43.2, SD 13.5 ^1^  (range 0 – 100) | 90-95% with 800 participants | 500 | 0.05 | 3 |  |
| **Patient satisfaction** | 20% | 800 | Mean 3.75, SD 1  (out of 5 – Likert scale)  ^2^ | >95% with 800 participants | 44 | 0.05 | 0.75 |  |
| **Knowledge score T1** | 20% | 800 | Mean 5.71, SD 1.55  (out of a possible 10) ^3^ | >95% with 800 participants | 30 | 0.05 | 1.40 |  |

# Supplementary Table 3: Sensitivity and subgroup analyses

## Genetic test uptake

|  | | Category representation (all participants) | | subgroup#intervention interaction | | | | Intervention effect in subgroup | | |
| --- | --- | --- | --- | --- | --- | --- | --- | --- | --- | --- |
|  |  | n | % | estimate | 95% CI | p-value | model n | estimate | 95% CI | p-value |
| Reported breast cancer history in first degree relatives | No | 789 | 74.57 | Reference category | | | | *No effect observed* | | |
|  | Yes | 269 | 25.43 | 0.62 | 0.18 to 2.18 | 0.455 | 1058 |  |  |  |
|  | Total | 1058 | 100.00 |  | | | |  |  |  |
| Reported breast cancer history in second degree relatives | No | 707 | 66.82 | Reference category | | | | *No effect observed* | | |
|  | Yes | 351 | 33.18 | 0.34 | 0.09 to 1.34 | 0.124 | 1058 |  |  |  |
|  | Total | 1058 | 100.00 |  | | | |  |  |  |
| Reported other cancer history in relatives | No | 886 | 83.74 | Reference category | | | | *No effect observed* | | |
|  | Yes | 172 | 16.26 | 0.47 | 0.07 to 3.22 | 0.445 | 1058 |  |  |  |
|  | Total | 1058 | 100.00 |  | | | |  |  |  |
| Breast cancer status | Newly diagnosed | 667 | 58.61 | Reference category | | | | *No effect observed* | | |
|  | In follow up | 400 | 35.15 | 1.41 | 0.64 to 3.07 | 0.392 | 1138 |  |  |  |
|  | Metastatic | 71 | 6.24 | 0.44 | 0.10 to 1.98 | 0.286 | 1138 |  |  |  |
|  | Total | 1138 | 100.00 |  | | | |  |  |  |
| Genetic test result | Negative | 969 | 97.00 | Not modelled | | | | | | |
|  | Pathogenic | 30 | 3.00 |  |  |  |  |  |  |  |
|  | Total | 999 | 100.00 |  |  |  |  |  |  |  |
| Method of receiving result from genetic test | Digitally | 942 | 94.29 | Not modelled | | | | | | |
|  | Telephone appointment | 57 | 5.71 |  |  |  |  |  |  |  |
|  | Total | 999 | 100.00 |  |  |  |  |  |  |  |

## Knowledge

|  | | | Category representation (all participants) | | | subgroup#intervention interaction | | | | Intervention effect in subgroup | | |
| --- | --- | --- | --- | --- | --- | --- | --- | --- | --- | --- | --- | --- |
|  |  |  | n | | % | estimate | 95% CI | p-value | model n | estimate | 95% CI | p-value |
| Reported breast cancer history in first degree relatives | No | 789 | | 74.57 | | Reference category | | | | *No effect observed* | | |
|  | Yes | 269 | | 25.43 | | -0.45 | -1.17 to 0.28 | 0.225 | 989 |  | | |
|  | Total | 1058 | | 100.00 | |  | | | |  | | |
| Reported breast cancer history in second degree relatives | No | 707 | | 66.82 | | Reference category | | | | *No effect observed* | | |
|  | Yes | 351 | | 33.18 | | 0.23 | -0.44 to 0.90 | 0.496 | 989 |  | | |
|  | Total | 1058 | | 100.00 | |  | | | |  | | |
| Reported other cancer history in relatives | No | 886 | | 83.74 | | Reference category | | | | *No effect observed* | | |
|  | Yes | 172 | | 16.26 | | 0.74 | -0.12 to 1.59 | 0.093 | 989 |  | | |
|  | Total | 1058 | | 100.00 | |  | | | |  | | |
| Breast cancer status | Newly diagnosed | 667 | | 58.61 | | Reference category | | | | *No effect observed* | | |
|  | In follow up | 400 | | 35.15 | | 0.30 | -0.38 to 0.98 | 0.382 | 989 |  | | |
|  | Metastatic | 71 | | 6.24 | | 0.45 | -0.92 to 1.82 | 0.520 |  |  |  |  |
|  | Total | 1138 | | 100.00 | |  | | | |  | | |
| Test result | Negative | 969 | | 97.00 | | Reference category | | | | *No effect observed* | | |
|  | Pathogenic | 30 | | 3.00 | | 1.20 | -0.64 to 3.05 | 0.202 | 987 |  | | |
|  | Total | 999 | | 100.00 | |  | | | |  | | |
| Method of receiving result | Digitally | 942 | | 94.29 | | Reference category | | | | *No effect observed* | | |
|  | Telephone appointment | 57 | | 5.71 | | 0.77 | -0.58 to 2.12 | 0.266 | 987 |  | | |
|  | Total | 999 | | 100.00 | |  | | | |  | | |

## Anxiety

|  | | Category representation (all participants) | | | subgroup#intervention interaction | | | | Intervention effect in subgroup | | |
| --- | --- | --- | --- | --- | --- | --- | --- | --- | --- | --- | --- |
|  |  | n | % | estimate | | 95% CI | p-value | model n | estimate | 95% CI | p-value |
| Reported breast cancer history in first degree relatives | No | 789 | 74.57 | Reference category | | | | | *No effect observed* | | |
|  | Yes | 269 | 25.43 | 0.13 | | -2.00 to 2.25 | 0.908 | 995 |  | | |
|  | Total | 1058 | 100.00 |  | | | | |  | | |
| Reported breast cancer history in second degree relatives | No | 707 | 66.82 | Reference category | | | | | *No effect observed* | | |
|  | Yes | 351 | 33.18 | -0.09 | | -2.05 to 1.87 | 0.929 | 995 |  | | |
|  | Total | 1058 | 100.00 |  | | | | |  | | |
| Reported other cancer history in relatives | No | 886 | 83.74 | Reference category | | | | | *No effect observed* | | |
|  | Yes | 172 | 16.26 | -0.34 | | -2.83 to 2.15 | 0.79 | 995 |  | | |
|  | Total | 1058 | 100.00 |  | | | | |  | | |
| Breast cancer status | Newly diagnosed | 667 | 58.61 | Reference category | | | | | *No effect observed* | | |
|  | In follow up | 400 | 35.15 | -2.10 | | -4.07 to -0.12 | 0.038 | 995 |  | | |
|  | Metastatic | 71 | 6.24 | -1.73 | | -5.74 to 2.28 | 0.399 |  |  |  |  |
|  | Total | 1138 | 100.00 |  | | | | |  | | |
| Test result | Negative | 969 | 97.00 | Reference category | | | | | *No effect observed* | | |
|  | Pathogenic | 30 | 3.00 | -1.06 | | -6.43 to 4.30 | 0.698 | 993 |  | | |
|  | Total | 999 | 100.00 |  | | | | |  | | |
| Method of receiving result | Digitally | 942 | 94.29 | Reference category | | | | | *No effect observed* | | |
|  | Telephone appointment | 57 | 5.71 | -0.56 | | -4.51 to 3.38 | 0.779 | 993 |  | | |
|  | Total | 999 | 100.00 |  | | | | |  | | |

## Satisfaction

|  | | Category representation (all participants) | | subgroup#intervention interaction | | | | Intervention effect in subgroup | | |
| --- | --- | --- | --- | --- | --- | --- | --- | --- | --- | --- |
|  |  | n | % | estimate | 95% CI | p-value | model n | estimate | 95% CI | p-value |
| Reported breast cancer history in first degree relatives | No | 789 | 74.6 | Reference category | | | | *No effect observed* | | |
|  | Yes | 269 | 25.4 | -0.14 | -o.34 to 0.07 | 0.198 | 908 |  | | |
|  | Total | 1058 | 100.0 |  | | | |  | | |
| Reported breast cancer history in second degree relatives | No | 707 | 66.8 | Reference category | | | | *No effect observed* | | |
|  | Yes | 351 | 33.2 | 0.06 | -0.12 to 0.24 | 0.511 | 908 |  | | |
|  | Total | 1058 | 100.0 |  | | | |  | | |
| Reported other cancer history in relatives | No | 886 | 83.7 | Reference category | | | | *No effect observed* | | |
|  | Yes | 172 | 16.3 | 0.10 | -0.15 to 0.34 | 0.441 | 908 |  | | |
|  | Total | 1058 | 100.0 |  | | | |  | | |
| Breast cancer status | Newly diagnosed | 667 | 58.6 | Reference category | | | | *No effect observed* | | |
|  | In follow up | 400 | 35.2 | 0.10 | -0.10 to 0.29 | 0.330 | 908 |  | | |
|  | Metastatic | 71 | 6.2 | -0.02 | -0.43 to 0.38 | 0.909 | 908 |  |  |  |
|  | Total | 1138 | 100.0 |  | | | |  | | |
| Test result | Negative | 969 | 97.0 | Reference category | | | | *No effect observed* | | |
|  | Pathogenic | 30 | 3.0 | -0.28 | -0.80 to 0.24 | 0.292 | 908 |  | | |
|  | Total | 999 | 100.0 |  | | | |  | | |
| Method of receiving result | Digitally | 942 | 94.3 | Reference category | | | | *No effect observed* | | |
|  | Telephone appointment | 57 | 5.7 | -0.21 | -0.59 to 0.17 | 0.276 | 908 |  | | |
|  | Total | 999 | 100.0 |  | | | |  | | |

# Supplementary table 4: Uptake of genetic testing by participant demographics

|  | Did not consent to genetic testing | | Consented to genetic testing (n=1,001) | | Overall (n=1,140) | |
| --- | --- | --- | --- | --- | --- | --- |
|  | n | % | n | % | n | % |
| **Age** |  |  |  |  |  |  |
| 18-30 years old | 0 | 0.0 | 1 | 0.1 | 1 | 0.1 |
| 31-40 years old | 3 | 2.2 | 54 | 5.4 | 57 | 5.0 |
| 41-50 years old | 20 | 14.4 | 223 | 22.3 | 243 | 21.3 |
| 51-60 years old | 37 | 26.6 | 316 | 31.6 | 353 | 31.0 |
| 61-70 years old | 34 | 24.5 | 261 | 26.1 | 295 | 25.9 |
| 71-80 years old | 33 | 23.7 | 122 | 12.2 | 155 | 13.6 |
| 81+ years old | 12 | 8.6 | 24 | 2.4 | 36 | 3.2 |
| Total *(with available data)* | 139 | 100.0 | 1001 | 100.0 | 1140 | 100.0 |
| Missing | 0 | 0.0 | 0 | 0.0 | 0 | 0.0 |
|  |  |  |  |  |  |  |
| **Ethnicity** |  |  |  |  |  |  |
| White | 47 | 83.9 | 837 | 84.1 | 884 | 84.1 |
| Asian or Asian British | 6 | 10.7 | 61 | 6.1 | 67 | 6.4 |
| Black African/Caribbean or Black British | 2 | 3.6 | 33 | 3.3 | 35 | 3.3 |
| Mixed or multiple ethnicities | 0 | 0.0 | 28 | 2.8 | 28 | 2.7 |
| Other | 1 | 1.8 | 36 | 3.6 | 37 | 3.5 |
| Total *(with available data)* | 56 | 40.3 | 995 | 99.4 | 1051 | 92.2 |
| Missing | 83 | 59.7 | 6 | 0.6 | 89 | 7.8 |
|  |  |  |  |  |  |  |
| **Highest qualification** |  |  |  |  |  |  |
| no qualifications | 4 | 7.7 | 67 | 6.9 | 71 | 7.0 |
| GCSE or equivalent | 16 | 30.8 | 205 | 21.2 | 221 | 21.7 |
| NVQ or equivalent | 4 | 7.7 | 129 | 13.4 | 133 | 13.1 |
| A Levels | 5 | 9.6 | 80 | 8.3 | 85 | 8.3 |
| Degree | 14 | 26.9 | 316 | 32.7 | 330 | 32.4 |
| Higher degree | 9 | 17.3 | 169 | 17.5 | 178 | 17.5 |
| Total *(with available data)* | 52 | 37.4 | 966 | 96.5 | 1018 | 89.3 |
| Missing | 87 | 62.6 | 35 | 3.5 | 122 | 10.7 |
|  |  |  |  |  |  |  |
| **Marriage status** |  |  |  |  |  |  |
| single | 11 | 19.6 | 121 | 12.3 | 132 | 12.7 |
| married or partnered | 28 | 50.0 | 683 | 69.4 | 711 | 68.4 |
| divorced | 7 | 12.5 | 106 | 10.8 | 113 | 10.9 |
| widowed | 10 | 17.9 | 74 | 7.5 | 84 | 8.1 |
| Total *(with available data)* | 56 | 40.3 | 984 | 98.3 | 1040 | 91.2 |
| Missing | 83 | 59.7 | 17 | 1.7 | 100 | 8.8 |
|  |  |  |  |  |  |  |
| **Employment status** |  |  |  |  |  |  |
| unemployed | 5 | 9.4 | 67 | 7.0 | 72 | 7.1 |
| working part time | 6 | 11.3 | 216 | 22.4 | 222 | 21.8 |
| working full time | 20 | 37.7 | 341 | 35.4 | 361 | 35.5 |
| retired | 22 | 41.5 | 340 | 35.3 | 362 | 35.6 |
| Total *(with available data)* | 53 | 38.1 | 964 | 96.3 | 1017 | 89.2 |
| Missing | 86 | 61.9 | 37 | 3.7 | 123 | 10.8 |

# References

1 Mansel RE, Fallowfield L, Kissin M et al. Randomized multicenter trial of sentinel node biopsy versus standard axillary treatment in operable breast cancer: the ALMANAC Trial. J Natl Cancer Inst 2006; 98 (9): 599-609.

2 Ware JE, Jr., Hays RD. Methods for measuring patient satisfaction with specific medical encounters. Medical care 1988; 26 (4): 393-402.

3 Meisel SF, Freeman M, Waller J et al. Impact of a decision aid about stratified ovarian cancer risk-management on women’s knowledge and intentions: a randomised online experimental survey study. BMC public health 2017; 17 (1): 882.
